# Comparative Genetics of Seed Size Traits in Divergent Cereal Lineages Represented by Sorghum (Panicoidae) and Rice (Oryzoidae)

**DOI:** 10.1534/g3.115.017590

**Published:** 2015-03-31

**Authors:** Dong Zhang, Jingping Li, Rosana O. Compton, Jon Robertson, Valorie H. Goff, Ethan Epps, Wenqian Kong, Changsoo Kim, Andrew H. Paterson

**Affiliations:** * Plant Genome Mapping Laboratory, University of Georgia, Athens, Georgia 30602; † Institute of Bioinformatics, University of Georgia, Athens, Georgia 30602; ‡ Department of Crop and Soil Sciences and Department of Genetics, University of Georgia, Athens, Georgia 30602; § Department of Plant Biology, University of Georgia, Athens, Georgia 30602

**Keywords:** quantitative trait locus, genome-wide association studies

## Abstract

Seed size is closely related to fitness of wild plants, and its modification has been a key recurring element in domestication of seed/grain crops. In sorghum, a genomic and morphological model for panicoid cereals, a rich history of research into the genetics of seed size is reflected by a total of 13 likelihood intervals determined by conventional QTL (linkage) mapping in 11 nonoverlapping regions of the genome. To complement QTL data and investigate whether the discovery of seed size QTL is approaching “saturation,” we compared QTL data to GWAS for seed mass, seed length, and seed width studied in 354 accessions from a sorghum association panel (SAP) that have been genotyped at 265,487 SNPs. We identified nine independent GWAS-based “hotspots” for seed size associations. Targeted resequencing near four association peaks with the most notable linkage disequilibrium provides further support of the role(s) of these regions in the genetic control of sorghum seed size and identifies two candidate causal variants with nonsynonymous mutations. Of nine GWAS hotspots in sorghum, seven have significant correspondence with rice QTL intervals and known genes for components of seed size on orthologous chromosomes. Identifying intersections between positional and association genetic data are a potentially powerful means to mitigate constraints associated with each approach, and nonrandom correspondence of sorghum (panicoid) GWAS signals to rice (oryzoid) QTL adds a new dimension to the ability to leverage genetic data about this important trait across divergent plants.

Seed size is a key trait in most plants (Moles *et al.* 2005) and is closely related to fitness, ecology, and domestication. Larger seeds contain more nutrition and are easier to harvest and process for human uses, but in the wild they are harder to disperse, sometimes germinate later, and penetrate soil poorly. Small seed size is sometimes associated with weediness (Susko *et al.* 2000). Grain size/mass was a primary target in the domestication of many crops, whereas relative allocation of resources to grain compared with biomass (harvest index) has accounted for much ongoing progress in crop yield improvement (Miller and Kebede 1984).

The Sorghum genus has recently become an important botanical model for Andropogoneae grasses by virtue of its relatively small and largely sequenced genome, a minimum of gene duplication because of 70 million years of abstinence from polyploidy, and its close relationship to grasses such as maize, sugarcane, and Miscanthus that have much more complex genomes (Paterson *et al.* 2009). Cultivated sorghum (*Sorghum bicolor*) ranks fifth in importance among the world’s grain crops and is a versatile source of food, fodder, and fuel. Seed size varies widely in this genus (Figure 1), and QTL mapping has been widely applied to uncover genomic regions that encode genes underlying this variation. However, QTL mapping is constrained to assessment of only those alleles that differ between the (usually two) parents of the study population; thus, determining the genetic complexity of a trait in a gene pool requires more information. Further, QTL mapping generally offers relatively coarse resolution that is seldom sufficient for identification of causative genes.

**Figure 1:**
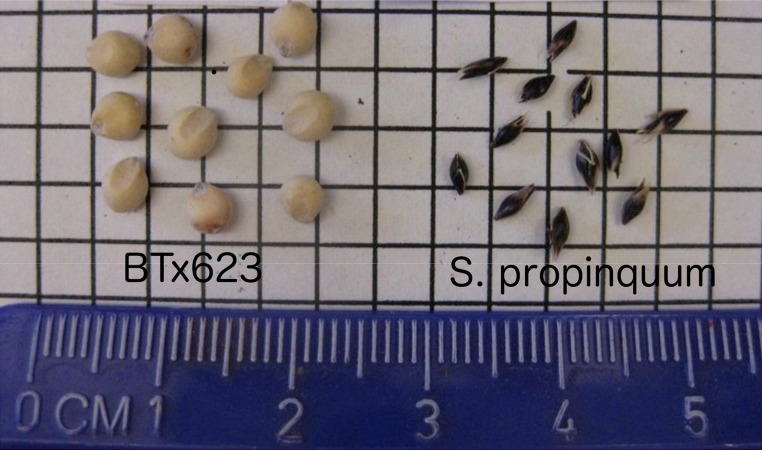
Comparison of seed size. *S. bicolor* genotype “BT×623” (which provided the reference sequence) and *S. propinquum* (which now has 30× Illumina sequence).

In contrast to QTL mapping, the accumulation of historical recombination events in long-term breeding populations or natural populations enables association mapping to improve the genetic resolution at which causative variants can be identified (Mackay *et al.* 2009). However, association mapping has constraints such as detection of spurious associations due to population structure (especially in improved germplasm), although statistical control and cautious experimental design can reduce the number of false-positive signals [*e.g.* (Thornsberry *et al.* 2001; Yu *et al.* 2006; Yu *et al.* 2008; Myles *et al.* 2009; Morris *et al.* 2012)]. Association mapping also has poor power to detect rare variants (minor allele frequency ≤0.05) of large effect (Morrell *et al.* 2011). The power to detect rare variants of intermediate to high effect is good using linkage approaches.

Several prior QTL studies (detailed below) are available for sorghum. Further, we have previously suggested that there exists nonrandom correspondence in the locations of genes conferring corresponding traits in divergent cereals (Paterson *et al.* 1995), and rich QTL data are available for seed size variation in another grass model, rice, with several of the underlying genes cloned (Fan *et al.* 2006; Song *et al.* 2007; Shomura *et al.* 2008; Wang *et al.* 2008; Weng *et al.* 2008; Li *et al.* 2012; Qi *et al.* 2012; X. Zhang *et al.* 2012). In the present study, QTL identified in four previous studies provide “prior evidence” implicating some genomic regions in the genetic control of seed size in sorghum. Identification of intersections among QTL, genome-wide association studies (GWAS), and comparative data advance knowledge of the genetic determinants of seed size variation in sorghum and provide a finer-scale comparison of the genetics of this important trait between sorghum and rice. Nonrandom correspondence of sorghum GWAS signals to rice QTL, *i.e.*, between divergent panicoid and oryzoid grasses, adds a new dimension to evidence of the ability to leverage genetic data about this important trait across divergent plants.

## Materials and Methods

### Genotyping for GWAS

We used 354 accessions from a U.S. sorghum association panel (SAP) (Casa *et al.* 2008) to perform GWAS. A total of 265,487 SNPs based on genotyping-by-sequencing (GBS) in 27,412 annotated genes were used (Morris *et al.* 2012). Approximately 72% of the genes contain ≥1 SNP site. A total of 228 of the 354 accessions are converted tropical lines that are photoperiod insensitive, early maturing, and short stature phenotypes produced via crossing exotic lines and modern U.S. cultivars (Casa *et al.* 2008). It has been demonstrated (Morris *et al.* 2012) that the population has sufficient power to dissect a trait, such as inflorescence architecture, that was not a target of selection in the sorghum conversion program.

### Targeted resequencing

Using GWAS, we chose four regions (see “Candidate genes in sorghum showing both QTL and GWAS evidence for seed size effects” for details) of the genome showing strong evidence of association and exhibiting strong linkage disequilibrium, three of which coincide with locations influencing seed size as determined by QTL mapping. Based on published genotypes, we found that all four association peaks are located in intergenic regions. For each hotspot, we selected two genes immediately flanking the association peak. For each gene candidate, one intron and one exon, both of which are at the side of the gene closer to the association peak, in 354 accessions from the SAP were sequenced. Detailed information on the PCR primers can be found in Supporting Information, Table S6. Resequencing used BigDye terminator chemistry, and the chromatograms were examined using SEQUENCHER software (version 4.1; GENECODES).

### Phenotype

Phenotypic data from three different growouts were utilized. On seed from Lubbock, Texas, grown during 2008, we measured the average mass, length, and width of 50 seeds before planting, which were named “2008 seed mass,” “2008 seed length,” and “2008 seed width.” We planted and evaluated the sorghum diversity panel in 2009 and 2010, near Watkinsville, Georgia, counting and weighing all seed from two representative heads per genotype to assess seed mass in each year (“2009 seed mass” and “2010 seed mass”). Measured parameters for seed size traits of sorghum are archived in Table S1.

### QTL mapping

We compiled 1-LOD likelihood intervals, which have been identified to underlie seed size traits from published literature. Of the four studies (Paterson *et al.* 1995; Brown *et al.* 2006; Feltus *et al.* 2006; Srinivas *et al.* 2009) included, one interspecific and two intraspecific, sorghum populations from eight different growouts were utilized to map seed size QTL. Specifically, a recombinant inbred line (RIL) from an interspecific cross between *S. bicolor* accession BT×623 and *S. propinquum* (BT×SP), the widest cross that can be made with *S. bicolor* using conventional techniques, containing 2512 loci on 10 linkage groups was used to map likelihood intervals for seed size traits (Paterson *et al.* 1995; Feltus *et al.* 2006); Brown *et al.* (2006) and Feltus *et al.* (2006) used two independent intraspecific genetic maps that were derived from two parental lines, *S. bicolor* accession IS3620C (IS) and BT×623, and consist of ∼3000 AFLP/RFLP/SSR markers (Brown *et al.* 2006) and 145 SSR/RFLP markers (Feltus *et al.* 2006), respectively; and a RIL set derived from an intraspecific cross between *S. bicolor* accession 296B and IS18551 was utilized (Srinivas *et al.* 2009). The methods for anchoring QTL intervals to the reference genome have been published (Zhang *et al.* 2013). Briefly, based on colinearity between genetic and physical positions of markers, a QTL region is delineated by two flanking markers nearest to the likelihood peak that have alignment information (BLASTN hits). The likelihood intervals for sorghum and rice seed size are archived in Table S2 and Table S5, respectively.

### Association analyses

The compressed mixed linear model (CMLM) involves genetic marker–based kinship matrix modeling of random effects used jointly with population structure estimated by principal components analysis (PCA) to model fixed effects (Bradbury *et al.* 2007; Zhang *et al.* 2010; Lipka *et al.* 2012). The compression level and optimal number of principal components that adequately explain population structure were previously determined by the Genomic Association and Prediction Integrated Tool (Lipka *et al.* 2012). Log quantile–quantile (QQ) *P* value plots for 265,487 single SNP tests of association (Figure S1) implied that there were few systematic sources of spurious association using CMLM, noting the close adherence of *P* values to the null hypothesis over most of the range.

### Significance threshold

A major issue with inferring statistically significant associations in genome scans is multiple testing. For example, 265,487 hypothesis tests were conducted in our studies of each trait. To adjust significance criteria to an experiment-wise *P* value of 0.05 for type I error (false positive), the Bonferroni method was used. For 265,487 tests, the significance cutoff for an overall probability of 0.05 for type I error can be approximated as 0.05/265,487 = 1.89 × 10^−7^. However, the Bonferroni correction is criticized for its stringency, reducing power to detect true associations, because some SNPs are correlated and thus are not truly independent hypotheses (The Wellcome Trust Case Control Consortium 2007). We performed Bonferroni-like multiple testing correction (Matthies *et al*. 2014) to determine significance thresholds for GWAS. Instead of 265,487 independent tests assumed in the Bonferroni method, the total number of tests was estimated by using the average extent of LD across the genome. On average, LD decays to background levels (*r*^2^ < 0.1) within 150kb in the current GBS data (Morris *et al*. 2012). The effective number of independent tests was defined as LD bins [reference genome size (730Mb)/average LD extent (150kb)]. Given 0.05 as the desired experiment wide probability of type I error, a significance cutoff within about an order of magnitude of 10^−5^ was estimated.

### Hotspot determination

Identifying intersections between positional and association genetic data is a potentially powerful means to mitigate constraints associated with each approach, accelerating progress toward identifying specific genes that function in biological processes of relevance to agriculture. In general, hotspots refer to eight genomic regions (Table S3) that have been implicated in linkage studies to underlie seed size traits and show strong GWAS-based association, which is defined by regions containing significant association SNPs (*P* ≤ 10^−5^) linked (*r^2^* ≥ 0.5) with ≥10 minor significant association (10^−5^ < *P* ≤ 10^−3^) markers for boundary setting, with seed size traits in our studies. One exceptional hotspot, albeit where no linkage studies have mapped, in telomeric regions (∼61.1 Mb) of chromosome Sb06 is strongly associated with seed mass and seed length in our studies.

### Genetic overlap evaluation

Fisher’s exact test was applied to assess overlap between QTL and GWAS within sorghum and to evaluate genetic correspondence between sorghum and rice. Within sorghum, the null hypothesis is that the proportion of GWAS-based associations (*P* ≤ 10^−5^ given by the CMLM) at loci within the QTL prior intervals is explicable by chance. The contingency table used in the test includes four categories: association loci within the QTL intervals; association loci outside the QTL intervals; nonassociation loci within the QTL intervals; and nonassociation loci outside the QTL intervals. Rejecting the null hypothesis supports nonrandom overlap between QTL and GWAS. Across taxa, Fisher’s exact test was used to test whether there is a random or nonrandom relationship between a sorghum GWAS association region and rice QTL intervals on their orthologous chromosomes. The test sample consists of genes having collinear orthologs in sorghum and rice and can be divided into four categories: genes within both sorghum association region and rice QTL intervals; genes within sorghum association region but outside rice QTL intervals; genes within rice QTL intervals but outside sorghum association region; and genes outside both the sorghum association region and rice QTL intervals. Finally, correspondence between sorghum and rice QTL was evaluated based on the proportions of sorghum QTL likelihood intervals within rice QTL intervals *vs.* outside rice QTL intervals. The Fisher’s exact test was used to determine the likelihood that the observed correspondence could be explained by chance.

### Reference genomes

The gene annotations refer to JGI annotation release Sbi1.4 (Paterson *et al.* 2009) and Michigan State University Rice Genome Annotation Project (MSU-RGAP release 7) (Kawahara *et al.* 2013).

## Results

### Race-specific patterns in seed size variation

**Figure 2:**
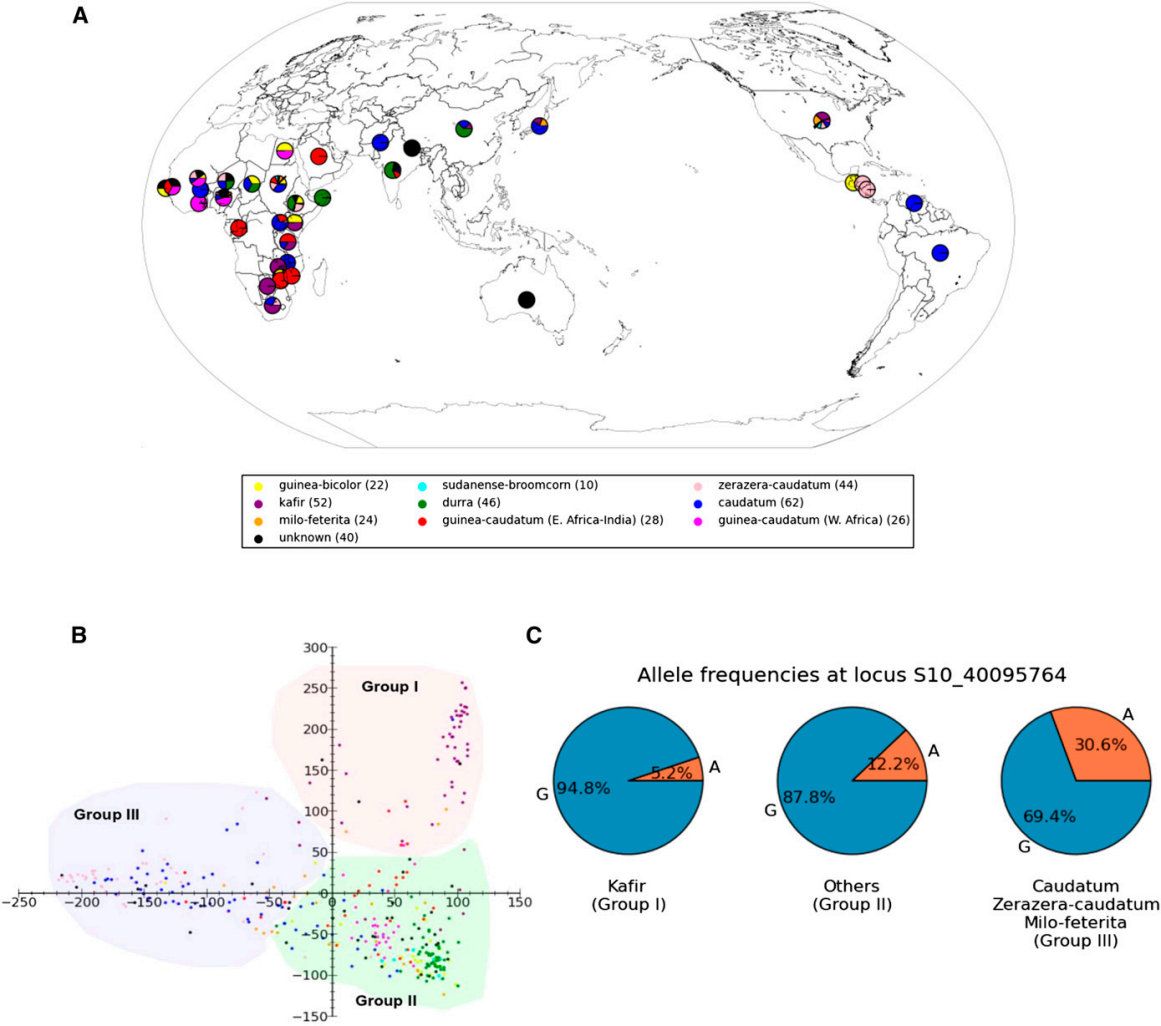
Germplasm origin and population structure of 354 accessions in a U.S. sorghum association panel (Casa *et al.* 2008). (A) Geographic origins of 354 sorghum accessions, color-coded by morphological types. A pie chart illustrates proportions of morphology at a location. (B) PCA plots of the first two components for 265,487 SNPs. Three color-coded subgroups for 354 sorghum accessions determined by K-means clustering. (C) The spectrum of allele frequencies at the SNP site (S10_40095764) characterized in gene *Sb10g018720*.

Geographic origins and domestication history can result in patterns of phenotypic variation among genotypes within a gene pool. We investigated whether phenotypic data for components of seed size exhibited variation patterns correlated with phylogenetic models for sorghum. The population structure of 354 accessions that broadly sample taxonomic, geographic, and morphological variations in cultivated forms of *Sorghum bicolor* (Casa *et al.* 2008) was determined from 265,487 SNPs by using principal component analysis (PCA) (Figure 2B). The 354 accessions were clustered into three subgroups by applying K-means clustering to the first two components of the PCA result (Figure 2B). Group I consists mostly of Kafir-type sorghums originally from southern Africa, which show a distinct genetic pattern relative to other races (Morris *et al.* 2012). The seeds of Kafir are considered to be medium in size (Magness *et al.* 1971). Group III is composed of most Caudatum, Zerazera-caudatum, and Milo-feterita types. Caudatum-type sorghums are generally considered to originate from central Africa and have large seeds. Milo and feterita types are found in northeast Africa and generally produce very large seeds (Magness *et al.* 1971). The remaining sorghum botanical races form group II. Based on *t*-tests (Table 1), group I and group III differ significantly in 2008 seed mass and seed width (with group III being larger and wider), but not in seed length. However, the variations are not observed in 2009 and 2010 seed mass data. Group II exhibits intermediate values of seed size traits and does not show significant seed size differences with the other two groups.

**Table 1:** Analysis of phenotypic differentiation among subgroups for seed size traits

		Group III	Group II
**Seed mass (2008)**	Group I	5.633 × 10^−5^*^a^*	0.1614
	Group II	0.003228	—
**Seed mass (2009)**	Group I	0.1336	0.7128
	Group II	0.3241	—
**Seed mass (2010)**	Group I	0.1494	0.8845
	Group II	0.1515	—
**Seed length (2008)**	Group I	0.02921	0.459
	Group II	0.08578	—
**Seed width (2008)**	Group I	1.213 × 10^−9^*^a^*	0.06561
	Group II	0.01608	—
**Seed length/width (2008)**	Group I	1.52 × 10^−8^*^a^*	0.1633
	Group II	0.0003824	—

*P* values shown were determined by t-test.

a Differentiation is significant at *P* ≤ 0.0001.

### Sorghum seed size QTL mapped in four genetic studies

QTL mapping is based on the principle that genes and linked DNA markers largely co-segregate during meiosis except for occasional recombination events, thus allowing their analysis in the progeny. The limited number of recombination events captured in progeny of recent crosses may result in QTL likelihood intervals that contain dozens or even hundreds of genes. Further, the environment and parental lines used in a cross can limit the power to accurately estimate the number and size of QTL.

Compilation of QTL mapping results from different parental combinations and in different environments yields a more complete picture of the genetic control of a trait than any single study (Rong *et al.* 2007). A total of 13 seed size QTL likelihood intervals published in four studies (Paterson *et al.* 1995; Brown *et al.* 2006; Feltus *et al.* 2006; Srinivas *et al.* 2009) fall into 11 nonoverlapping regions in the sorghum genome, strongly implicating that genetic control of sorghum seed size involves at least 11 genes (Zhang *et al.* 2013) (Table S2). The genetic basis of seed size was explored in one interspecific cross and two intraspecific crosses studied under a total of eight different environments. Thus, the four mapping studies were able to capture many genetic variations for seed size. Of the 13 QTL likelihood intervals identified, phenotypic variation explained in their original mapping population ranged from 4.8 to 14.8%. A comparison across the four independent studies demonstrates that only two pairs of QTL intervals are co-localized on chromosome Sb03 [Paterson *et al.* 1995; Feltus *et al.* 2006 (SB×SP cross)] and Sb06 (Srinivas *et al.* 2009; Feltus *et al.* 2006), respectively. The relatively large proportion of noncorresponding QTL intervals may reflect that seed size can be influenced by a relatively large number of genes with modest phenotypic effects, also interacting with the environment.

**Figure 3:**
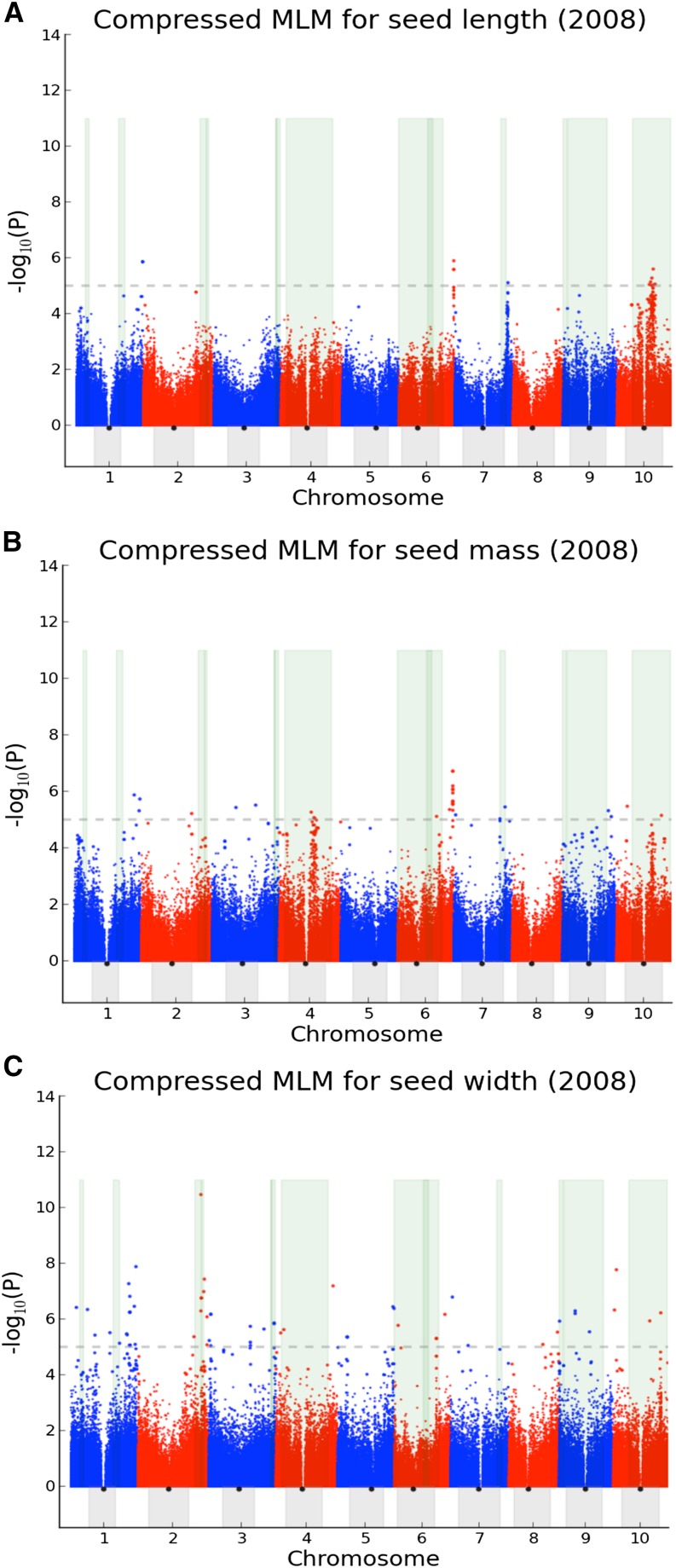
Genome-wide association studies of seed length, seed mass, and seed width in 2008. (A) Manhattan plot of compressed mixed linear model (CMLM) (see *Materials and Methods* for details) for 2008 seed length. The 10 sorghum chromosomes are plotted against the negative base-10 logarithm of the association *P* value, with significance threshold denoted by the gray dashed line. The areas highlighted in green indicate likelihood intervals for seed size determined by QTL mapping. Heterochromatin and centromeres are indicated by the gray areas and black dots, respectively. (B) Manhattan plot of CMLM for 2008 seed mass. (C) Manhattan plot of CMLM for 2008 seed width.

For functional loci located in the pericentromeric region, the general lack of recombination can allow QTL likelihood intervals to cross centromeres and to cover broad genomic areas. Of the 13 QTL intervals for seed size, four cross centromeres (Table S2 and Figure 3). Mapped QTL intervals tend to have finer resolution in euchromatin, where there is more recombination generally, such as the intervals identified on chromosomes Sb02 and Sb07 (Figure 3). Rapidly growing genetic/genomic data may provide future information to discern whether additional QTL affecting seed size in sorghum are either consistent in multiple genotypes or specific to single genotypes.

### GWAS for three seed size–related traits

**Figure 4:**
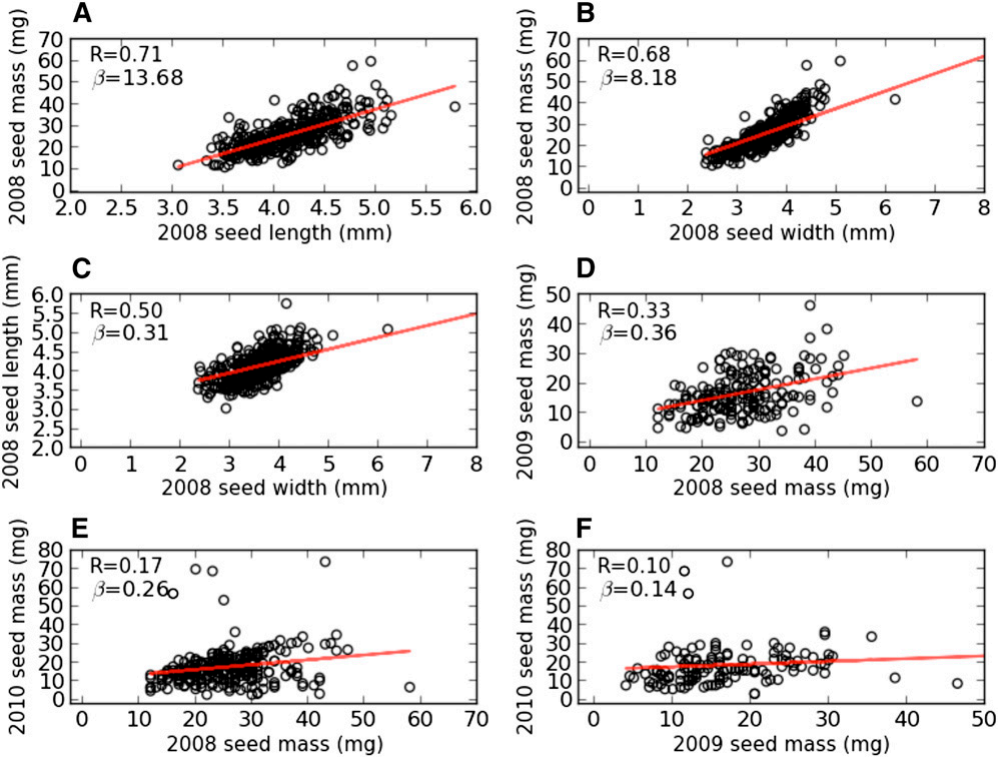
Relationships among seed size–related traits from 354 accessions in a U.S. sorghum association panel (Casa *et al.* 2008). Red lines indicate linear regression resulting from least squares fitting, with Pearson correlation coefficients (R) and the slopes of the linear regression (β) shown. (A) The correlation between 2008 seed mass and 2008 seed length. (B) The correlation between 2008 seed mass and 2008 seed width. (C) The correlation between 2008 seed width and 2008 seed length. (D) The correlation between 2008 seed mass and 2009 seed mass. (E) The correlation between 2008 seed mass and 2010 seed mass. (F) The correlation between 2009 seed mass and 2010 seed mass.

To further investigate the genetic basis of seed size in sorghum, we conducted GWAS on seed mass (measured in the SAP grown in 3 yr at two locations), seed length, and seed width (each measured in a single year and location) (Figure 3 and Figure S2). GWAS results detected with SAP nonrandomly overlap (*P* = 0.125 × 10^−7^) (see the testing of overlaps in the *Materials and Methods*) with QTL intervals. To characterize the relationship among seed size–related traits, the Pearson correlation coefficient is calculated for each pair of traits. Strong correlations are observed for seed length, width, and mass in 2008 (Figure 4, A–C). However, low heritability (0.31) derived from seed mass in 2008, 2009, and 2010 and weak phenotypic correlations across years (Figure 4, D–F) indicate a strong effect of environment on seed size traits. The slopes of the regression lines (Figure 4, A and B) reflect that seed mass changes more with one-unit changes in seed length than in seed width.

**Figure 5:**
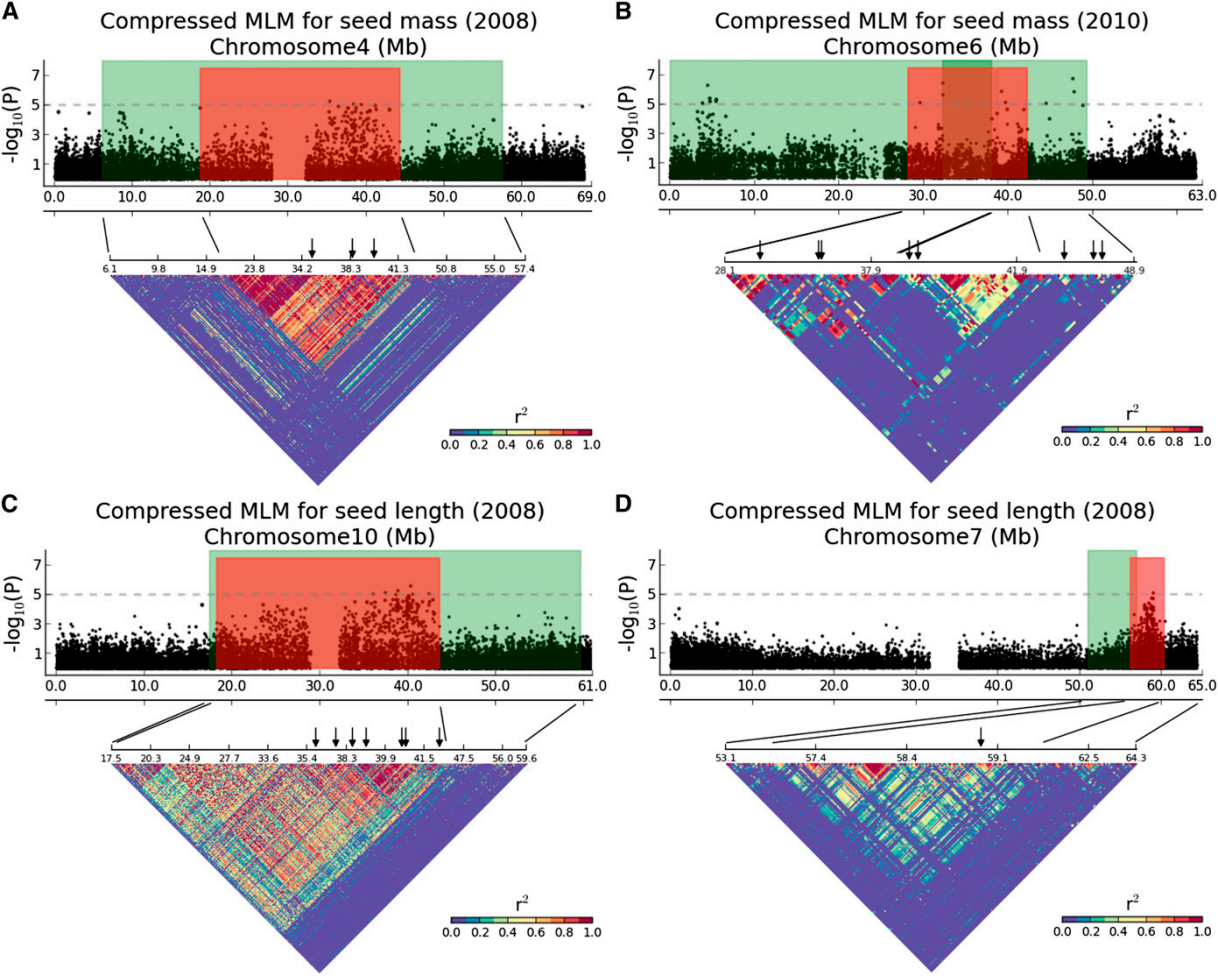
Chromosome-wide Manhattan plots (top) for seed size traits. Green areas indicate likelihood intervals for seed size determined by QTL mapping. Red areas show hotspot for seed size identified by association mapping. Linkage disequilibrium (*r*^2^) matrices (bottom) are plotted for regions denoted by anchoring lines. Regions of strong LD are shown in red. Significant association markers are denoted by black arrows. (A) The 2008 seed mass associations on chromosome Sb04. (B) The 2010 seed mass associations on chromosome Sb06. Another association hotspot (chr Sb06: 2129372-5985416) is denoted in Table S3. (C) The 2008 seed length associations on chromosome Sb10. (D) The 2008 seed length associations on chromosome Sb07.

For GWAS applied to 2008 seed length data (Figure 3A), there are three association peaks collectively accounting for 54% of phenotypic variation. One of these (Table S3) is in the centromeric region of chromosome Sb10 and two (Table S3) are in telomeric regions of chromosomes Sb06 and Sb07. All three regions exhibit clear LD block patterns (Figure 5, C and D and Figure S3B), even though the two euchromatic association hotspots have relatively weak and short LD blocks. Two of these association peaks, on chromosomes Sb07 and Sb10 (Figure 5, C and D), coincide closely with likelihood intervals (Table S2) identified by QTL mapping in two sorghum populations: *S. bicolor* L. Moench accession BT×623 (BT) × *S. propinquum* (SP) consisting of 370 F2 progeny and BT × *S. bicolor* accession IS3620C (IS) consisting of 137 F6-8 RILs (Feltus *et al.* 2006). Some discrepancy between the association peak and the likelihood interval on chromosome Sb07 may be due to statistical limitations of QTL mapping or uneven coverage of genetic maps. The association hotspot (Figure S3B) on chromosome Sb06 is also associated with 2008 seed mass (Figure S3A).

One additional apparent association “skyline” (Figure 3B) explaining 31% of phenotypic variation in 2008 seed mass was in the heterochromatin of chromosome Sb04, which shows a clear LD block (Figure 5A) and is supported by a prior QTL likelihood interval (Table S2) in the BT×SP cross (Paterson *et al.* 1995). The map resolution of the QTL was refined from ∼51.4 Mb based on QTL mapping (Table S2) to ∼25.6 Mb based on GWAS (Table S3).

For 2008 seed width data, most association loci are distributed in euchromatin, with locally rapid LD decay. For example, one identified hotspot (Figure S3C) close to the 5′ end of chromosome Sb09 explains 13% of phenotypic variation in 2008 seed width and co-localizes with one QTL likelihood interval from BT×IS3620C. The map resolution of the QTL was refined from ∼4 Mb based on QTL mapping (Table S2) to ∼70,000 bases based on GWAS (Table S3).

### Candidate genes in sorghum showing both QTL and GWAS evidence for seed size effects

Linkage disequilibrium, a property that has long been of interest in population genetics, is “nonrandom” association of alleles at different loci resulting from shared histories of mutation and recombination. For a trait of interest, association analysis has the potential to identify causative variants and their linked loci simultaneously. To investigate gene candidates for sorghum seed size, we further examined four genomic regions exhibiting notable LD and harboring multiple SNP sites strongly associated with seed size. Two of these regions are located in heterochromatin of chromosomes Sb04 and Sb10 (Figure 5, A and C), each detected by both GWAS and QTL mapping (Paterson *et al.* 1995; Feltus *et al.* 2006) and each of which significantly correspond to rice QTL for seed size (Li *et al.* 1997; Rabiei *et al.* 2004). Another region (Figure 5D) identified by both GWAS and QTL mapping (Feltus *et al.* 2006) is located in the telomeric region of sorghum chromosome Sb07. One region (Figure S3, A and B) extending from 61.1 Mb to 61.4 Mb on sorghum chromosome Sb06 is only identified by GWAS.

**Figure 6:**
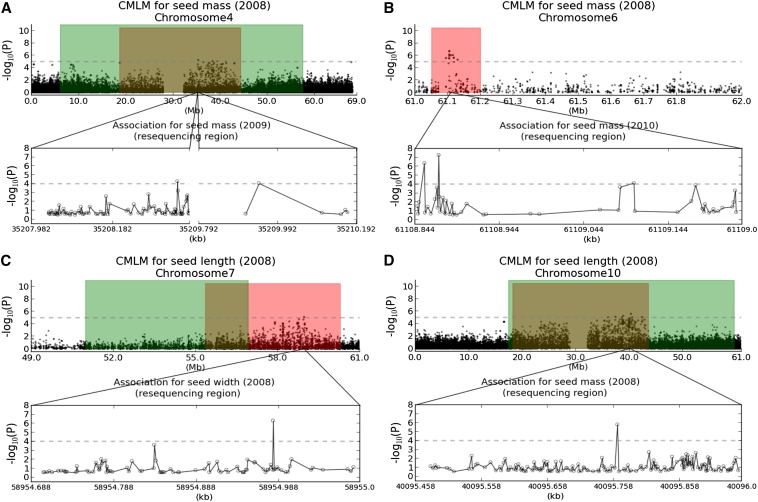
Strength of association for seed size traits in targeted resequencing regions. Green areas indicate likelihood intervals for seed size determined by QTL mapping. Red areas show hotspot for seed size identified by association mapping. Four candidate genes [(A) *Sb04g015420*, (B) *Sb06g033060*, (C) *Sb07g023950*, and (D) *Sb10g018720*] encode six variants that are strongly associated (*P* ≤ 10^−4^) with sorghum seed size.

Targeted resequencing of 354 accessions from a sorghum diversity panel (Casa *et al.* 2008) near the four strongest association peaks (Figure 6) provides further support of the role(s) of these regions in the genetic control of sorghum seed size and identifies two candidate causal variants. All four association peaks are located at intergenic loci based on the public reduced representation sequence (Morris *et al.* 2012). We investigated the two nearest genes flanking each of the four association peaks (listed in Table S6). For each gene candidate, we sequenced one intron and one exon (see details in the *Targeted resequencing*). For example, genes *Sb10g018720* (chr Sb10: 40,087,709-40,096,277) and *Sb10g018920* (chr Sb10: 40,584,355-40,586,470) are chosen for the intergenic locus S10_40270546 on chromosome Sb10. A total of 903 SNP sites in the eight genes flanking the four GWAS peaks were identified on the basis of sequence alignment. Of the eight genes evaluated, four [*Sb04g015420*, *Sb06g033060*, *Sb07g023950*, and *Sb10g018720* from JGI annotation release Sbi1.4 (Paterson *et al.* 2009)] encode six variants that are strongly associated with seed size (*P* ≤ 10^−4^) (Table S7). Two common variants [minor allele frequency (MAF) ≥5%] were observed in *Sb06g033060* (at nucleotide S6_61108872 in Sbi1.4) and *Sb10g018720* (at nucleotide S10_40095764), both of which contain nucleotide changes that can alter the amino acid sequence relative to the sorghum reference genome (Paterson *et al.* 2009). The substitutions for the other four polymorphic sites (S4_35209945, S4_35208341, S6_61108855, S7_58954981) are silent relative to the sorghum reference genome, noting that S6_61108855 is only 13 nt from candidate causal variant S6_61108872.

Gene *Sb10g018720*, specifically putative fiber protein Fb34, contains one nonsynonymous SNP site (S10_40095764) with MAF of 20%, which is significantly associated with 2008 seed mass (*P* = 1.57 × 10^−6^) and 2008 seed width (*P* = 9.6 × 10^−6^). *Sb10g018720* appears to be a homolog of a gene originally discovered based on expression in seed-borne epidermal fibers of cotton (Uniprot accession Q6T7D3). Allele distribution (Figure 2C) at locus S10_40095764 reflects phylogenetic patterns determined by PCA analysis (Figure 2B). Allele “A,” a substitution for “G” that alters the encoded amino acid from arginine (R) to lysine (K) relative to the sorghum reference genome (Paterson *et al.* 2009), is associated with increased seed size and accounts for 30.6% of alleles in group III (Caudatum, Zerazera-caudatum, and Milo-feterita), shown above to have heavier and wider seed size *vs.* only 5.2% in group I (Kafir) with smaller and narrower seed size (Table 1). Group II has intermediate allele frequency for allele “A” (12.2%) and also cannot be distinguished from either group I or group III based on seed size.

Another gene harboring a nonsynonymous SNP site (S6_61108872) is *Sb06g033060*, annotated as “similar to H0801D08.10 protein” and appearing to be a member of the major facilitator superfamily (MFS: Interpro IPR005828). This superfamily includes transmembrane proteins that are widespread in both prokaryotes and eukaryotes, single-polypeptide secondary carriers thought to transport small solutes in response to chemiosmotic ion gradients. Allele “C,” a substitution for “T” that alters the amino acid isoleucine (I) to threonine (T) relative to the sorghum reference genome (Paterson *et al.* 2009), is associated with heavier seeds but is evenly distributed in the three racial groups.

### Genetic correspondence in diverse cereals

Synteny and colinearity have been well-conserved between major grass clades such as the panicoids (represented by sorghum) and the oryzoids (represented by rice) since their divergence approximately 50 million years ago (Mya), enabling us to compare causal loci in corresponding regions across taxa. An early comparative QTL study found positional correspondence among seed mass (size) QTL in sorghum, rice, and maize at a frequency that was only explicable by chance in 0.1 to 0.8% of cases (Paterson *et al.* 1995), suggesting that there may be functionally conserved genomic regions that underlie seed size variation in diverse cereals. GWAS data provide an additional means to explore such correspondence (beyond QTL mapping), with its intrinsic advantage of higher resolution but also its disadvantage of more false-positive associations. Additionally, the common ancestor of these lineages experienced a whole-genome duplication (WGD; named rho) approximately 70 Mya that is still readily discernible in their genomes (Paterson *et al.* 2004), making it possible to further test the hypothesis that homeologous genomic regions still have some corresponding functions after 70 million years of divergence.

**Figure 7:**
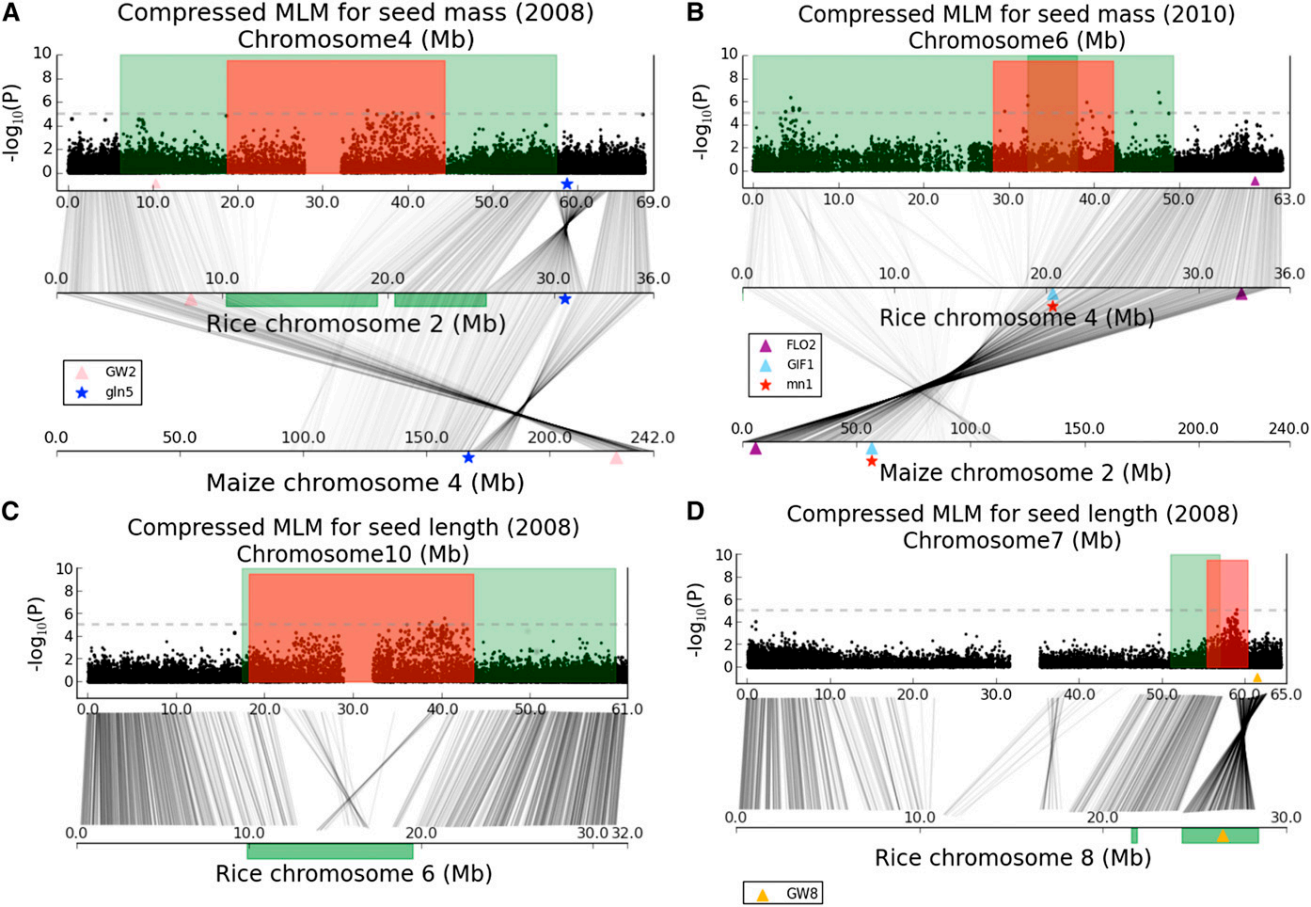
Genetic correspondence across sorghum, rice, and maize. Genomic regions implicated in sorghum seed size by association (red) and QTL likelihood intervals (green) are shown. Genomic regions implicated in rice seed size by QTL studies are denoted by green areas. The known seed size genes in rice and maize are indicated by color-coded triangles and stars, respectively. Gray connecting lines indicate pairs of duplicated genes. (A) Genetic correspondence on sorghum chromosome Sb04, rice chromosome Os02, and maize chromosome Zm04. (B) Genetic correspondence on sorghum chromosome Sb06, rice chromosome Os04, and maize chromosome Zm02. (C) Genetic correspondence on sorghum chromosome Sb10 and rice chromosome Os06. (D) Genetic correspondence on sorghum chromosome Sb07 and rice chromosome Os08.

Prior comparisons of the genetic control of corresponding traits in divergent taxa based on QTL data (Paterson *et al.* 1995) can now be enhanced with the addition of GWAS data, and in some cases also with genes that have been demonstrated to be causal of phenotypic variation. Evaluating correspondence between seed size GWAS hotspots in sorghum and QTL or candidate genes in rice may provide evidence of convergent domestication (Paterson *et al.* 1995) at higher resolution than QTL mapping. We are aware of 10 rice genes (Fan *et al*. 2006; Song *et al*. 2007; Shomura *et al*. 2008; Wang *et al*. 2008; Takano-Kai *et al*. 2009; Abe *et al*. 2010; Kitagawa *et al*. 2010; Mao *et al*. 2010; She *et al*. 2010; Li *et al*. 2011; Qi *et al*. 2012; Wang *et al*. 2012; X. Zhang *et al*. 2012) and three maize genes (Giroux *et al*. 1996; Gupta *et al*. 2006; Martin *et al*. 2006) (Table S4) that have been verified to function in seed size traits using map-based cloning. We used synteny, which strengthens the inference of paralogs or orthologs beyond a blast match, to locate the sorghum “homologs” for these 13 known genes. Although large-scale colinearity is shared between significant association intervals in sorghum and regions containing genes shown to be causal of seed size variation in rice, none of the sorghum homologs are encompassed in the identified hotpots. For example, two loci on rice chromosome Os04 have been identified to be involved in regulation of rice grain, *FLO2* (*FLOURY ENDOSPERM2*) (She *et al.* 2010) and *GIF1* (*GRAIN INCOMPLETE FILLING 1*) (Wang *et al.* 2008), in which *GIF1* is orthologous to *mn1* (*miniature1*) (Gupta *et al.* 2006) influencing the development of maize endosperm. We found that sorghum has experienced gene loss for *GIF1* on chromosome Sb06, which is orthologous to rice chromosome Os04 (Figure 7B). Moreover, the sorghum ortholog of rice *FLO2* is physically far away from the hotpots we found (Figure 7B). It appears likely that we mapped novel loci on sorghum chromosome Sb06 underlying seed size, rather than orthologs of *FLO2* and *GIF1*. Another case is that of *GW8* (*OsSPL16*) (Wang *et al.* 2012) on rice chromosome Os08, which encodes a protein that is a positive regulator of cell proliferation. The sorghum ortholog (chr Sb07: 61,426,227-61,420,520) of *GW8* is ∼2.5 Mb away from the association peak (chr Sb07: 58,953,828) (Figure 7D). It is likely that the association we found on sorghum chromosome Sb07 is related to a gene that is different from *GW8*. Two more instances of sorghum-rice-maize comparisons can be found on Sb04-Os02-Zm04 (Figure 7A) and on Sb03-Os01-Zm03 (Figure S4C). Rice gene *GW2* (Song *et al.* 2007), and maize genes *gln5* (Martin *et al.* 2006) and *Sh2* (Giroux *et al.* 1996) are not physically close to regions inferred to confer seed size variation in sorghum.

In addition to 10 known genes, we compiled 17 rice seed-size QTL likelihood intervals (Table S5). Of nine association hotspots in sorghum, four have significant correspondence with rice QTL intervals (see *Materials and Methods* for details of overlap testing) on orthologous chromosomes (Figure 7 and Figure S4). For example, the association signals detected in the pericentromeric regions of sorghum chromosome Sb04 and Sb10 share a large number of gene duplications with QTL intervals (Rabiei *et al.* 2004) identified in heterochromatin regions of rice chromosomes Os02 and Os06, respectively (Figure 7, A and C). The association signal located in the euchromatin of sorghum chromosome Sb07 shows a connection with two genomic regions implicated in linkage studies (Rabiei *et al.* 2004; Xie *et al.* 2006) on rice chromosome Os08. Such correspondence may reflect functionally conserved “genomic regions” existing across taxa, but may or may not be due to corresponding (orthologous or paralogous) genes. Genes in a pathway exhibit significantly higher genomic clustering than expected by chance in eukaryotes (Lee and Sonnhammer 2003); for example, co-regulated clusters of genes have been implicated in QTL affecting cotton fiber traits (Paterson *et al.* 2012).

A hypothesis worthy of further exploration is that a co-regulated cluster of genes in the cereal common ancestor may have experienced gain/loss and/or functional divergence of some members in the subsequent 70 million years, with independent domestications conferring additional functional changes in similar locations of different taxa but that are not strictly orthologous. Three notable cases are QTL likelihood intervals corresponding (Figure S4, B, C, and D) on sorghum chromosome Sb02 (Paterson *et al.* 1995; Feltus *et al.* 2006) and rice chromosome Os07 (Shao *et al.* 2010; Zhao *et al.* 2010), on Sb03 (Paterson *et al.* 1995; Feltus *et al.* 2006) and Os01 (Li *et al.* 1997; Thomson *et al.* 2003), and on Sb01 (Feltus *et al.* 2006; Srinivas *et al.* 2009) and Os03 (Bai *et al.* 2010), in which Os03 contains known cloned *GS3* (Fan *et al.* 2006; Takano-Kai *et al.* 2009; Mao *et al.* 2010) and *qGL3* (Qi *et al.* 2012; X. Zhang *et al.* 2012) at 16.7 Mb and 25.0 Mb, respectively. Although we did not find evidence for strong GWAS-based association in any of these sorghum QTL intervals within our studies, the correspondence suggests potentially interesting regions for seed size that can be further explored by using different association or fine-mapping strategies. We noted that the genetic maps used in Paterson *et al.* (1995) and Feltus *et al.* (2006) were derived from an interspecific cross between *S. bicolor* and *S. propinquum* (Figure 1), which are separated by 1–2 million years. Such wide crosses may have better statistical power to detect variants with low/rare frequency in intraspecific collections such as the sorghum-association panel (SAP).

## Discussion

Using seed size traits of sorghum, our studies demonstrate that GWAS can be used to improve the genetic resolution for likelihood intervals determined by QTL mapping. Genomic regions in which sorghum QTL have repeatedly been discovered in past studies provide us with compilations of prior information toward the goal of uncovering causative variants. Some GWAS studies have suggested that association mapping of inbreeding organisms may realize lower precision than outcrossing organisms (Morrell *et al.* 2011). Sorghum is largely inbreeding, which can result in strong LD patterns. We have shown two cases in the pericentromeric regions with low genetic resolution on chromosomes Sb04 and Sb10. The degree of improvement in resolution by GWAS over QTL mapping is related to the nature of the “genomic environment” surrounding a gene—with substantial improvement in recombinationally active euchromatin but much less improvement in recombinationally recalcitrant heterochromatin with long LD blocks. Compared to domesticated populations, wild populations that have experienced recombination for many thousands or more of reproductive cycles may be of great utility to achieve high resolution by association mapping (Huang *et al.* 2012).

It is important to determine LD with single SNP association, especially when causative variants are not genotyped (or at least are not known). In the SNP set used in this study (Morris *et al.* 2012), there are one or more variants within a gene genotyped for 72% of 27,412 high-confidence annotated genes in the reference genome sequence, which may cause a high probability of missing causative loci. Sorghum is largely inbreeding, which can result in strong LD patterns along the genome, and provides a rich source of haplotype blocks to localize genomic regions associated with causative genes for seed size. On the basis of pairwise measures of LD (*r*^2^), “block-like” structures can be visually apparent, tending to be longer in the pericentromeric region, which experiences relatively little recombination, than the euchromatin, which experiences more frequent recombination. A long LD block with association signals is most likely to contribute striking features to the “skyline” of a genome-wide Manhattan plot.

Association mapping and QTL mapping are complementary to each other. For example, some phenotype–genotype associations were found only in one of the two mapping strategies. In general, QTL mapping is based on differences between only two parental lines that often differ greatly in phenotype, such as *S. bicolor* and *S. propinquum*, which are separated by 1–2 million years. Such wide crosses may segregate for alleles that are not sampled in intraspecific collections such as the sorghum association panel (SAP). Another example is a classical maturity locus in sorghum, *Ma3/phyB*, which was tentatively identified (but not confirmed by mutant complementation) with a map-based strategy (Childs *et al.* 1997) but is wild-type in most sorghum cultivars and converted exotic lines, so significant association signals are not found (Morris *et al.* 2012).

Three genomic regions have been revealed to be necessary for temperate adaptation across all sorghum conversion lines containing the *Dwarf* (*Dw*)*1*, *Dw2*, and *Dw3* loci underlying sorghum plant height on chromosomes Sb09, Sb06, and Sb07, respectively (Lin *et al.* 1995; Morris *et al.* 2012; Thurber *et al.* 2013). Our study demonstrates that two LD blocks contain both dwarfing genes and association loci for seed size. On chromosome Sb07, *Dw3* (∼58.6 Mb) is very close to the association peak (∼58.9 Mb) for seed size, and the strong linkage disequilibrium (*r*^2^ = 0.6) between the two association peaks indicates that their inheritance is linked either functionally (pleiotropy) or physically (linkage disequilibrium). In contrast, on chromosome Sb06, there is a relatively big gap and very weak linkage disequilibrium (*r*^2^ = 0.08) between *Dw2* (∼42 Mb) and the association peak (∼39 Mb) for seed size. So, it is more likely that *Dw2* and the seed size loci on Sb06 merely co-locate in a large heterochromatic block but are not otherwise closely related.

Our comparative studies across taxa suggest that large-scale homeologous segments preserve functional regions affecting seed size traits in sorghum, rice, and maize. Numerous studies have indicated that orthologs across taxa have similar functions underlying common phenotypes, but quite a few genes have no obvious counterparts in their close species. Hence, whether specific conserved “genes” are responsible for genetic variation in seed size in both sorghum and rice is still a question mark. The relatively large genomic distance between the likelihood peaks of seed size associations and the locations of sorghum paralogs/orthologs of known rice seed size genes suggests that sorghum-specific loci are most likely identified in our GWAS.

Genotyping-by-sequencing, at present still generally using reduced representation approaches to be economical, provides both proxy DNA markers and a foundation for identifying gene candidates that warrant further investigation by targeted resequencing. By partially resequencing genes, we identified four candidates containing six mutations that showed a strong association with the target phenotype. Although only two of the alleles that we found altered amino acid composition relative to the sorghum reference genotype, the other four may be closely linked to causal mutations, perhaps in nearby regulatory DNA. Resequencing of entire genes and surrounding regulatory DNA may provide further insight into the specific functional mutations associated with phenotypic effects of these alleles. Curiously, some GWAS conducted in rice and *Arabidopsis thaliana* have suggested that known causative loci showed weaker signals than nearby proxy DNA markers (Morrell *et al.* 2011). Hence, resequencing genes surrounding the peak of association is not guaranteed to pinpoint the causative loci. One could envision engaging numerous additional data types beyond the QTL meta-data and comparative data that we have used here, for example, examining expression profiles in a particular tissue to further aid in the determination of candidate genes (Paterson *et al.* 2012).

Our data suggest that environmental factors are a large element of the answer to an important fundamental question in gene mapping, specifically why the GWAS approach has revealed so little variation (Manolio *et al.* 2009). Even given “perfect” (*i.e.*, 100% accurate) information about phenotype and genotype, some associations may not be repeatable due to interaction between genotype and environment (Ross-Ibarra *et al.* 2007). For example, seed size is correlated with the environmental conditions under which species establish (Moles *et al.* 2005). We found that seed mass data measured in years 2009 and 2010 failed to verify associations identified based on 2008 seed mass data (Figure S2). However, 2010 seed mass data show suggestive associations on chromosomes Sb05, Sb06, and Sb08 that were not found in prior years but that are supported by QTL data. In particular, two 2010 seed mass hotspots (Figure 5B) determined on chromosome Sb06 each correspond to QTL likelihood intervals (Table S2). Combined with associations inferred based on 2008 seed mass (Figure S3A), this suggests that there are three or more loci underlying seed mass on chromosome Sb06, the effects of which are likely to depend on the environment.

## Supplementary Material

Supporting Information
