# Supplementary material for: Comparative Genetics of Seed Size Traits in Divergent Cereal Lineages Represented by Sorghum (Panicoidae) and Rice (Oryzoidae)
Source: G3 (Bethesda). 2015 Mar 31;5(6):1117–28. doi: 10.1534/g3.115.017590 (PMC4478542; doi:10.1534/g3.115.017590)
Supplement: Supporting Information [file supp_5_6_1117__index.html]

Comparative Genetics of Seed Size Traits in Divergent Cereal Lineages Represented by Sorghum (Panicoidae) and Rice (Oryzoidae) — Supporting Information 

# Comparative Genetics of Seed Size Traits in Divergent Cereal Lineages Represented by Sorghum (Panicoidae) and Rice (Oryzoidae)

## Supporting Information for Zhang *et al.*, 2015

**Files in this Data Supplement:**

- Supporting Information - Figures S1-S4 and Tables S1-S7 (PDF, 672 KB)
- Figure S1 - Log quantile-quantile (QQ) of compressed MLM plots for 265,487 single-SNP tests of association. (PDF, 378 KB)
- Figure S2 - Genome-wide association studies of seed mass in 2009 and 2010. (PDF, 504 KB)
- Figure S3 - Chromosome-wide Manhattan plots (top) for seed size traits. (PDF, 575 KB)
- Figure S4 - Genetic correspondence across sorghum, rice and maize. (PDF, 564 KB)
- Table S1 - Measured parameters for seed size traits of sorghum. Accessions, trait names, years are indicated. (.xlsx, 25 KB)
- Table S2 - 1-LOD likelihood intervals for seed size traits of sorghum determined by QTL mapping. Trait names, genomic positions, flanking markers and references are indicated. (.xlsx, 10 KB)
- Table S3 - Hotspots for seed size traits of sorghum identified by GWAS. Trait names, genomic positions and peaks of association are indicated. (.xlsx, 10 KB)
- Table S4 - 13 published genes shown to be causal of seed size variation in rice and maize. Gene names, effect and references are listed. (.xlsx, 10 KB)
- Table S5 - 1-LOD likelihood intervals for seed size traits of rice determined by QTL mapping. Trait names, genomic positions, flanking markers and references are indicated. (.xlsx, 9 KB)
- Table S6 - Summary of PCR primers used for 8 gene candidates in targeted resequencing regions. (.xlsx, 9 KB)
- Table S7 - Significant association variants characterized in targeted resequencing regions for 4 sorghum gene candidates. Gene identifiers, genomic positions, association *P* values, alleles and MAF are indicated. (.xlsx, 9 KB)
