# Supplementary material for: Comparative Genetics of Seed Size Traits in Divergent Cereal Lineages Represented by Sorghum (Panicoidae) and Rice (Oryzoidae)
Source: G3 (Bethesda). 2015 Mar 31;5(6):1117–28. doi: 10.1534/g3.115.017590 (PMC4478542; doi:10.1534/g3.115.017590)
Supplement: Supporting Information [file supp_g3.115.017590_017590SI.pdf]

G3 · Genes | Genomes | Genetics

Comparative genetics of seed size traits in divergent cereal lineages represented by sorghum (Panicoidae) and rice (Oryzoidae)

Dong Zhang[*,§], Jingping Li[*,§], Rosana O. Compton[*], Jon Robertson[*], Valorie H. Goff[*], Ethan Epps[*], Wenqian Kong[*,‡], Changsoo Kim[*] and Andrew H. Paterson[*,§,†,‡,1]

.

[*] Plant Genome Mapping Laboratory, University of Georgia, Athens, GA 30602, USA

[§] Institute of Bioinformatics, University of Georgia, Athens, GA 30602, USA

[†] Department of Plant Biology, University of Georgia, Athens, GA 30602, USA

[‡] Department of Crop and Soil Sciences and Department of Genetics, University of Georgia, Athens, GA 30602, USA

[1]Corresponding author: Andrew H. Paterson
Address: 111 Riverbend Road, Rm 228, Athens, GA 30602
Tel: +1 706 583 0162
Fax: +1 706 583 0160
Email: paterson@.uga.edu

DOI: 10.1534/g3.115.017590

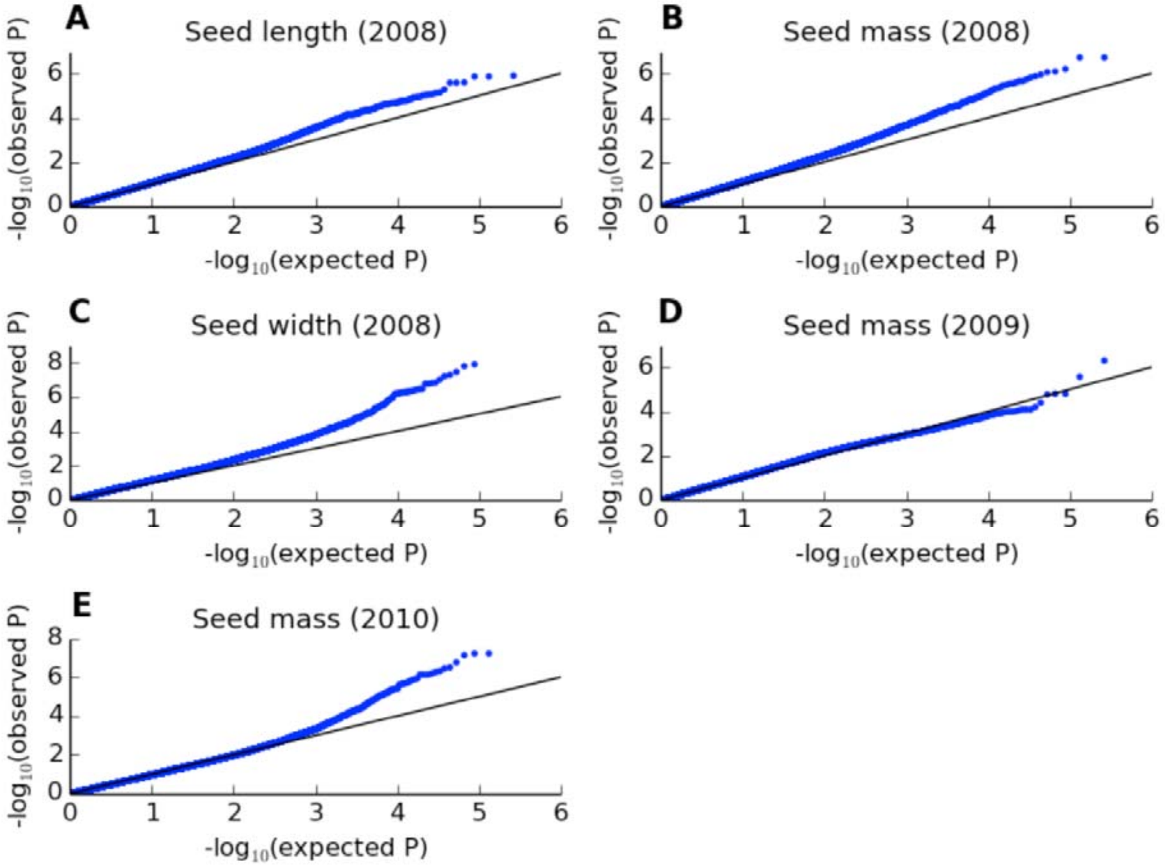

Figure S1 Log quantile–quantile (QQ) of compressed MLM plots for 265,487 single-SNP tests of association. The association *P* values are indicated by blue lines. The black lines correspond to the null hypotheses. (A) QQ plot for 2008 seed length. (B) QQ plot for 2008 seed mass. (C) QQ plot for 2008 seed width. (D) QQ plot for 2009 seed mass. (E) QQ plot for 2010 seed mass.

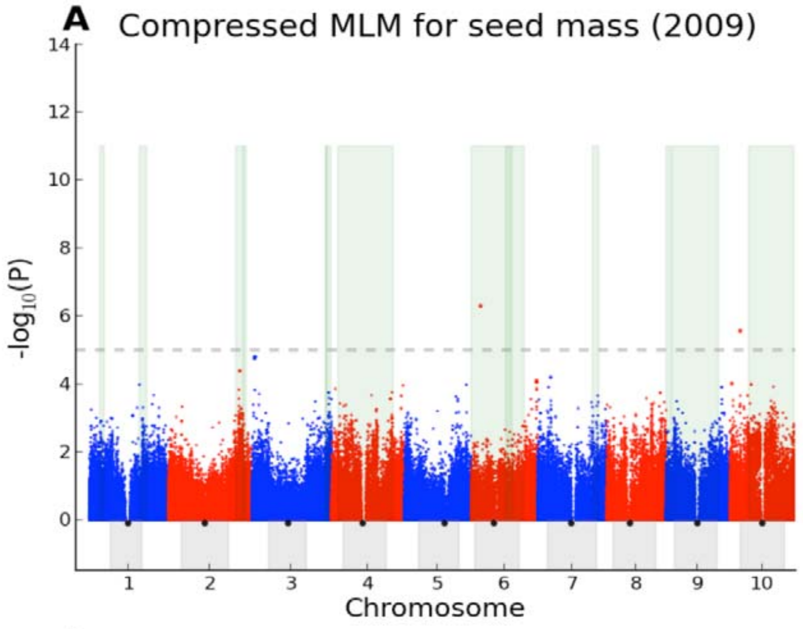

Figure S2 Genome-wide association studies of seed mass in 2009 and 2010. (A) Manhattan plot of CMLM for 2009 seed mass. Significance threshold is denoted by the gray dashed line. The 10 sorghum chromosomes are plotted against the negative base-10 logarithm of the association P value. The areas highlighted in green indicate confidence intervals for seed size determined by QTL mapping. Heterochromatin and centromeres are indicated by the gray areas and the black dots individually. (B) Manhattan plot of CMLM for 2010 seed mass.

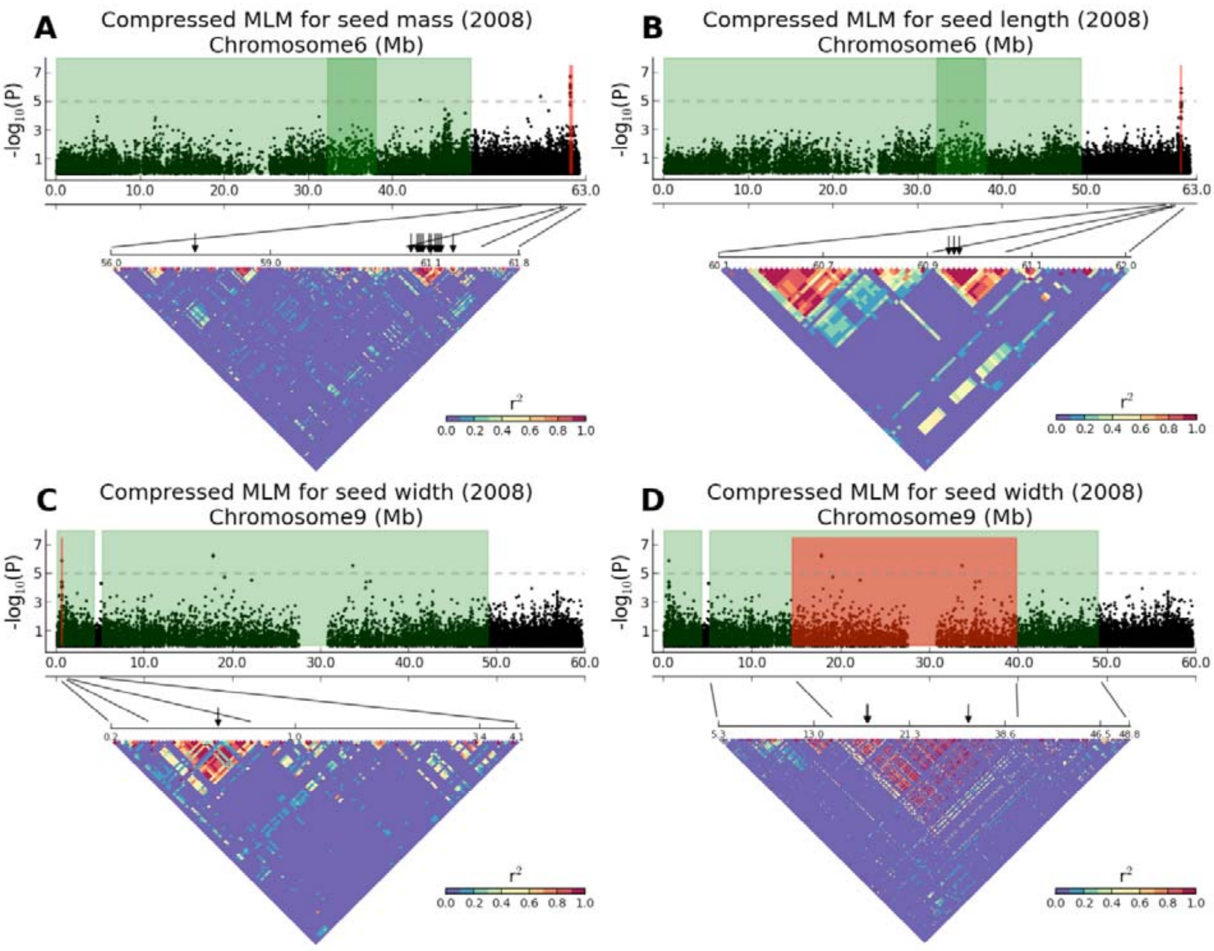

Figure S3 Chromosome-wide Manhattan plots (top) for seed size traits. Green areas indicate confidence intervals for seed size determined by QTL mapping. Red areas show hotspot for seed size identified by association mapping. Linkage disequilibrium (r^2) matrices (bottom) are plotted for regions denoted by anchoring lines. Regions of strong LD are shown in red. Significant association markers are denoted by black arrows. (A) 2008 seed mass associations on chromosome Sb06. (B) 2008 seed length associations on chromosome Sb06. (C) (D) 2008 seed width associations on chromosome Sb09.

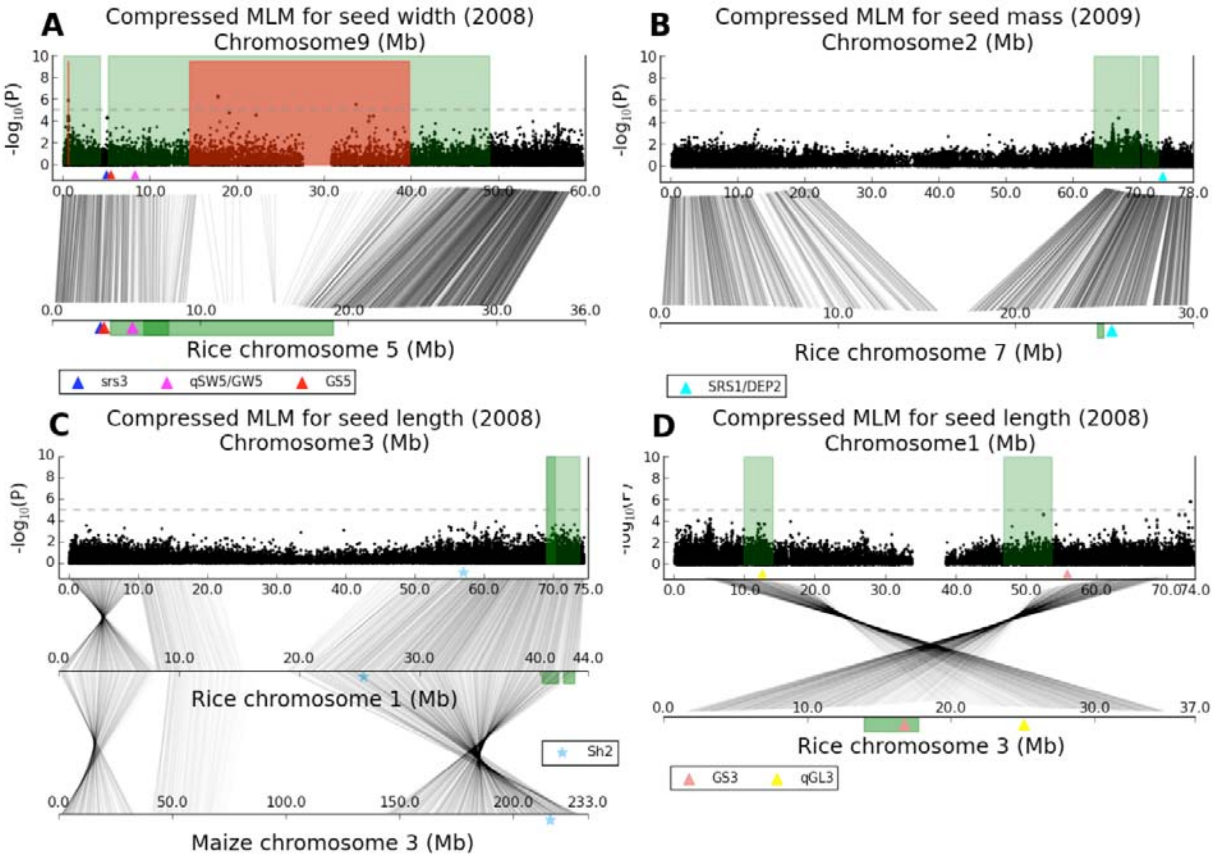

Figure S4 Genetic correspondence across sorghum, rice and maize. Hotspots identified in sorghum, with association regions highlighted in red and QTL confidence interval highlighted in green. Genomic regions implicated in linkage studies to affect rice seed size are denoted by green areas in rice. The known seed size genes in rice and maize are indicated by color-coded triangles and stars individually. Gray connecting lines indicate pairs of duplicated genes. (A) Genetic correspondence on sorghum chromosome Sb09 and rice chromosome Os05. (B) Genetic correspondence on sorghum chromosome Sb02 and rice chromosome Os07. (C) Genetic correspondence on sorghum chromosome Sb03, rice chromosome Os01 and maize chromosome Zm03. (D) Genetic correspondence on sorghum chromosome Sb01 and rice chromosome Os03.

Tables S1-S7

Available for download as Excel files at www.g3journal.org/lookup/suppl/doi:10.1534/g3.115.017590/-/DC1

Table S1 Measured parameters for seed size traits of sorghum. Accessions, trait names, years are indicated.

Table S2 1-LOD likelihood intervals for seed size traits of sorghum determined by QTL mapping. Trait names, genomic positions, flanking markers and references are indicated.

Table S3 Hotspots for seed size traits of sorghum identified by GWAS. Trait names, genomic positions and peaks of association are indicated.

Table S4 13 published genes shown to be causal of seed size variation in rice and maize. Gene names, effect and references are listed.

Table S5 1-LOD likelihood intervals for seed size traits of rice determined by QTL mapping. Trait names, genomic positions, flanking markers and references are indicated.

Table S6 Summary of PCR primers used for 8 gene candidates in targeted resequencing regions.

Table S7 Significant association variants characterized in targeted resequencing regions for 4 sorghum gene candidates. Gene identifiers, genomic positions, association *P* values, alleles and MAF are indicated.
