# Supplementary material for: Comparative Genetics of Seed Size Traits in Divergent Cereal Lineages Represented by Sorghum (Panicoidae) and Rice (Oryzoidae)
Source: G3 (Bethesda). 2015 Mar 31;5(6):1117–28. doi: 10.1534/g3.115.017590 (PMC4478542; doi:10.1534/g3.115.017590)
Supplement: Supporting Information [file supp_g3.115.017590_FigureS1.pdf]

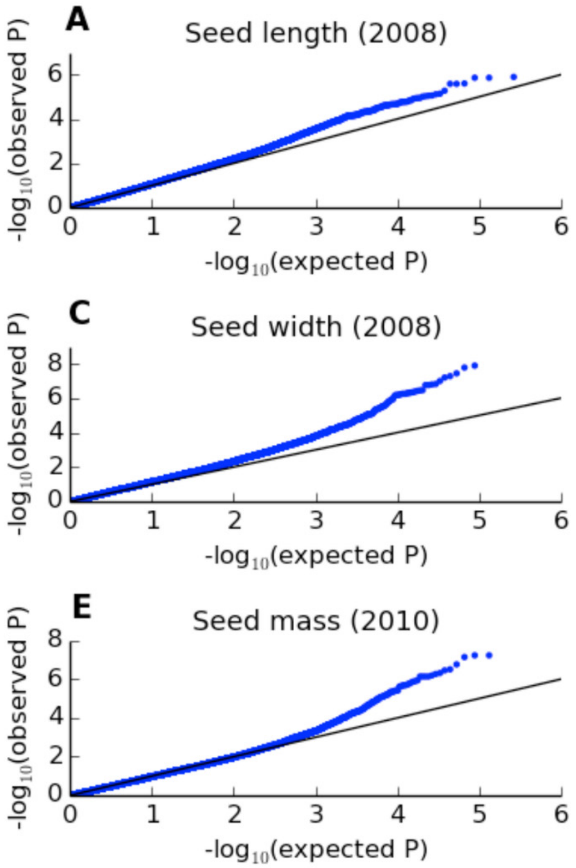

Figure S1 Log quantile–quantile (QQ) of compressed MLM plots for 265,487 single-SNP tests of association. The association *P* values are indicated by blue lines. The black lines correspond to the null hypotheses. (A) QQ plot for 2008 seed length. (B) QQ plot for 2008 seed mass. (C) QQ plot for 2008 seed width. (D) QQ plot for 2009 seed mass. (E) QQ plot for 2010 seed mass.
