# Supplementary material for: Comparative Genetics of Seed Size Traits in Divergent Cereal Lineages Represented by Sorghum (Panicoidae) and Rice (Oryzoidae)
Source: G3 (Bethesda). 2015 Mar 31;5(6):1117–28. doi: 10.1534/g3.115.017590 (PMC4478542; doi:10.1534/g3.115.017590)
Supplement: Supporting Information [file supp_g3.115.017590_FigureS2.pdf]

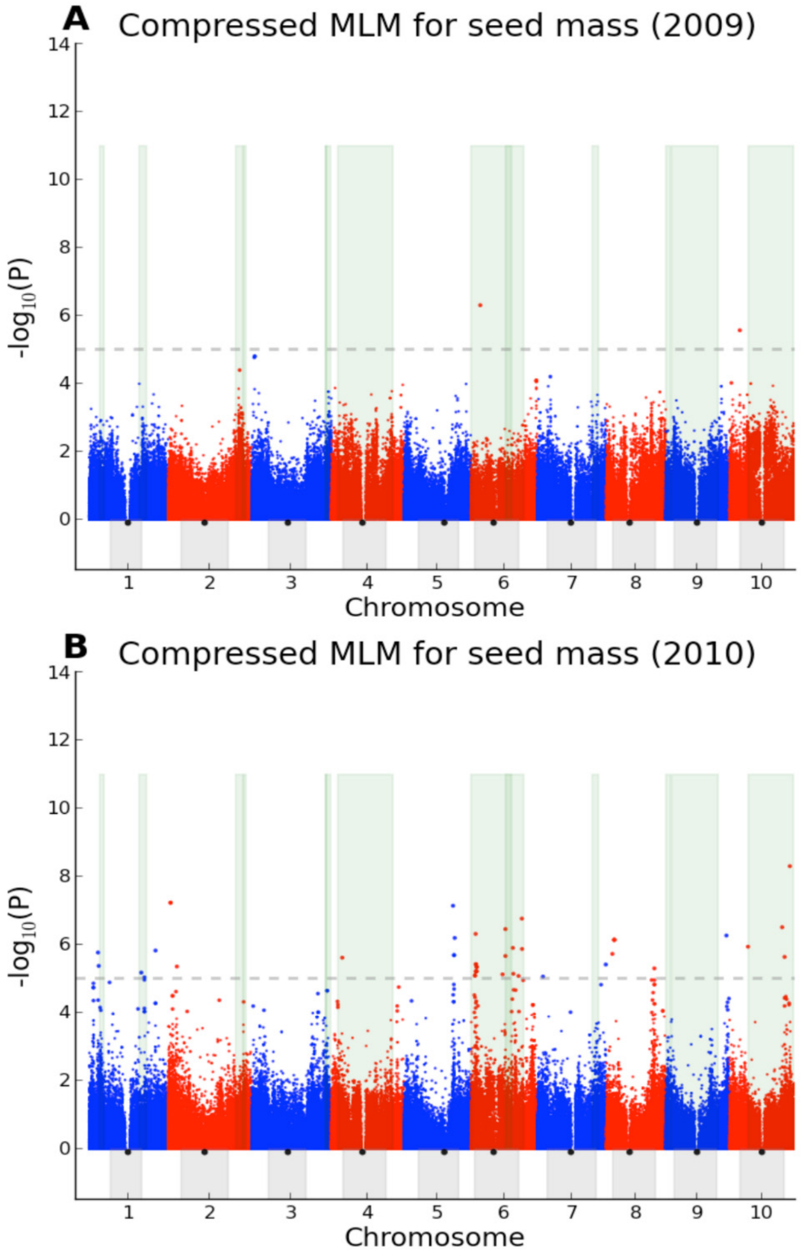

Figure S2 Genome-wide association studies of seed mass in 2009 and 2010. (A) Manhattan plot of CMLM for 2009 seed mass. Significance threshold is denoted by the gray dashed line. The 10 sorghum chromosomes are plotted against the negative base-10 logarithm of the association P value. The areas highlighted in green indicate confidence intervals for seed size determined by QTL mapping. Heterochromatin and centromeres are indicated by the gray areas and the black dots individually. (B) Manhattan plot of CMLM for 2010 seed mass.
