# Supplementary material for: Comparative Genetics of Seed Size Traits in Divergent Cereal Lineages Represented by Sorghum (Panicoidae) and Rice (Oryzoidae)
Source: G3 (Bethesda). 2015 Mar 31;5(6):1117–28. doi: 10.1534/g3.115.017590 (PMC4478542; doi:10.1534/g3.115.017590)
Supplement: Supporting Information [file supp_g3.115.017590_FigureS3.pdf]

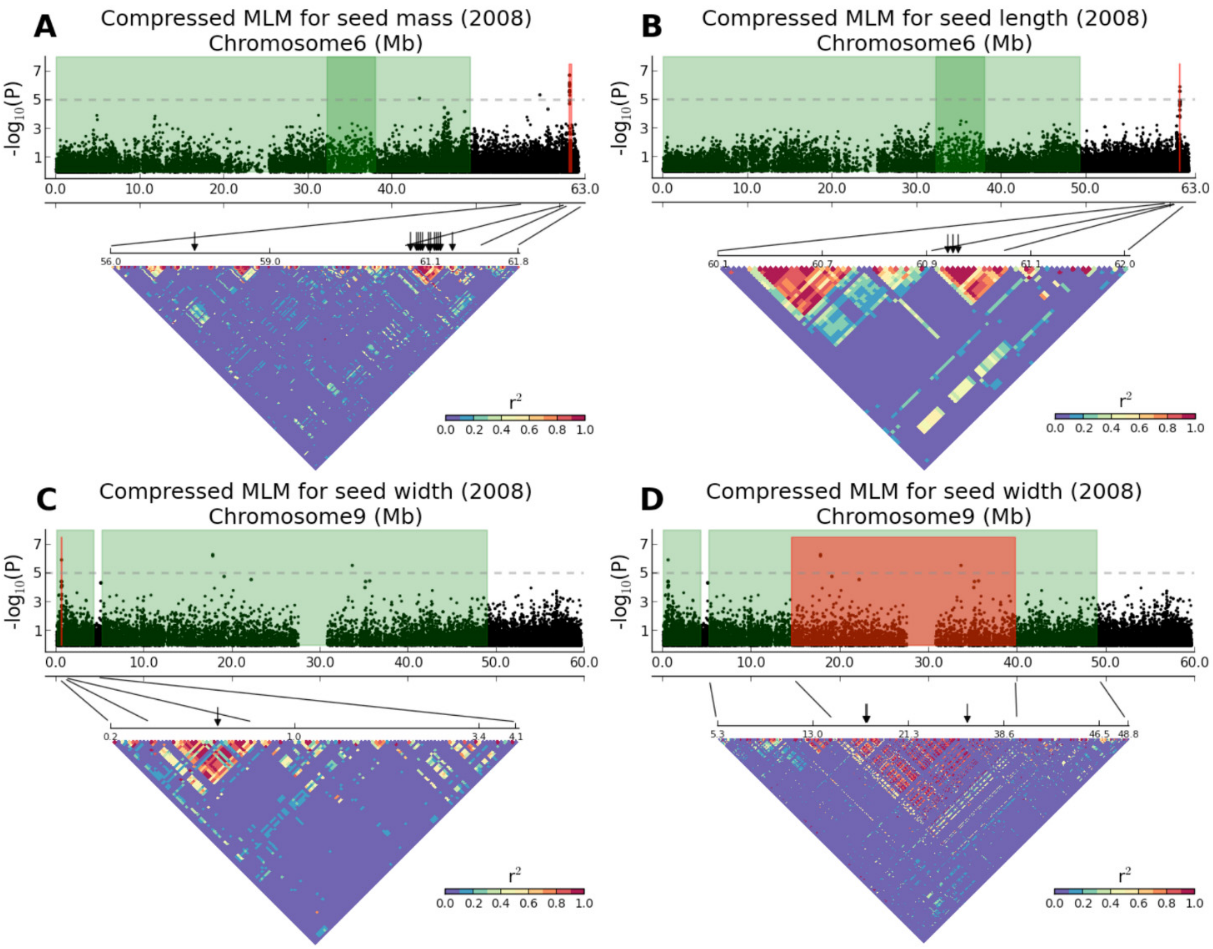

Figure S3 Chromosome-wide Manhattan plots (top) for seed size traits. Green areas indicate confidence intervals for seed size determined by QTL mapping. Red areas show hotspot for seed size identified by association mapping. Linkage disequilibrium (r^2) matrices (bottom) are plotted for regions denoted by anchoring lines. Regions of strong LD are shown in red. Significant association markers are denoted by black arrows. (A) 2008 seed mass associations on chromosome Sb06. (B) 2008 seed length associations on chromosome Sb06. (C) (D) 2008 seed width associations on chromosome Sb09.
