# Supplementary material for: Comparative Genetics of Seed Size Traits in Divergent Cereal Lineages Represented by Sorghum (Panicoidae) and Rice (Oryzoidae)
Source: G3 (Bethesda). 2015 Mar 31;5(6):1117–28. doi: 10.1534/g3.115.017590 (PMC4478542; doi:10.1534/g3.115.017590)
Supplement: Supporting Information [file supp_g3.115.017590_FigureS4.pdf]

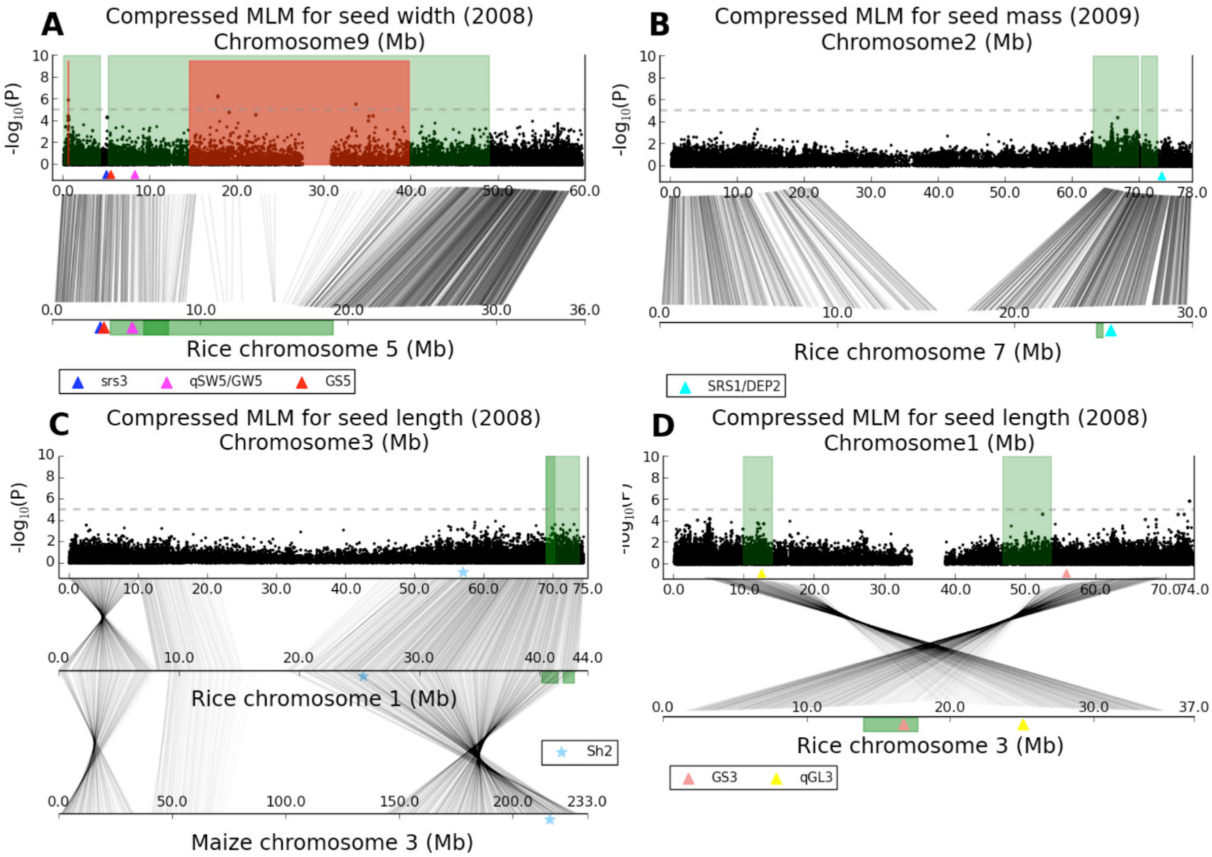

Figure S4 Genetic correspondence across sorghum, rice and maize. Hotspots identified in sorghum, with association regions highlighted in red and QTL confidence interval highlighted in green. Genomic regions implicated in linkage studies to affect rice seed size are denoted by green areas in rice. The known seed size genes in rice and maize are indicated by color-coded triangles and stars individually. Gray connecting lines indicate pairs of duplicated genes. (A) Genetic correspondence on sorghum chromosome Sb09 and rice chromosome Os05. (B) Genetic correspondence on sorghum chromosome Sb02 and rice chromosome Os07. (C) Genetic correspondence on sorghum chromosome Sb03, rice chromosome Os01 and maize chromosome Zm03. (D) Genetic correspondence on sorghum chromosome Sb01 and rice chromosome Os03.
